# Selenium Inhibits Root Elongation by Repressing the Generation of Endogenous Hydrogen Sulfide in *Brassica rapa*

**DOI:** 10.1371/journal.pone.0110904

**Published:** 2014-10-21

**Authors:** Yi Chen, Hai-Zhen Mo, Mei-Yu Zheng, Ming Xian, Zhong-Qiang Qi, You-Qin Li, Liang-Bin Hu, Jian Chen, Li-Fei Yang

**Affiliations:** 1 College of Horticulture, Nanjing Agricultural University, Nanjing, China; 2 Institute of Food Quality and Safety, Jiangsu Academy of Agricultural Sciences, Nanjing, China; 3 Department of Food Science, Henan Institute of Science and Technology, Xinxiang, Henan Province, China; 4 Lishui Plant Science Base, Jiangsu Academy of Agricultural Sciences, Nanjing, China; 5 Department of Chemistry, Washington State University, Pullman, Washington, United States of America; University of Missouri-Kansas City, United States of America

## Abstract

Selenium (Se) has been becoming an emerging pollutant causing severe phytotoxicity, which the biochemical mechanism is rarely known. Although hydrogen sulfide (H_2_S) has been suggested as an important exogenous regulator modulating plant physiological adaptions in response to heavy metal stress, whether and how the endogenous H_2_S regulates Se-induce phytotoxicity remains unclear. In this work, a self-developed specific fluorescent probe (WSP-1) was applied to track endogenous H_2_S *in situ* in the roots of *Brassica rapa* under Se(IV) stress. Se(IV)-induced root growth stunt was closely correlated with the inhibition of endogenous H_2_S generation in root tips. Se(IV) stress dampened the expression of most *LCD* and *DCD* homologues in the roots of *B. rapa*. By using various specific fluorescent probes for bio-imaging root tips *in situ*, we found that the increase in endogenous H_2_S by the application of H_2_S donor NaHS could significantly alleviate Se(IV)-induced reactive oxygen species (ROS) over-accumulation, oxidative impairment, and cell death in root tips, which further resulted in the recovery of root growth under Se(IV) stress. However, dampening the endogenous H_2_S could block the alleviated effect of NaHS on Se(IV)-induced phytotoxicity. Finally, the increase in endogenous H_2_S resulted in the enhancement of glutathione (GSH) in Se(IV)-treated roots, which may share the similar molecular mechanism for the dominant role of H_2_S in removing ROS by activating GSH biosynthesis in mammals. Altogether, these data provide the first direct evidences confirming the pivotal role of endogenous H_2_S in modulating Se(IV)-induced phytotoxicity in roots.

## Introduction

Selenium (Se) contamination is a global environmental safety issue because Se is becoming an emerging health hazards due to the dramatic increase in Se concentration in the environment [1], [2]. The rapid development of metal industry promotes the biogeochemical cycle of Se, which results in the remarkably anthropogenic release of Se into soil [1], [3]. Se is an essential micronutrient for plants because Se-containing proteins play vital roles in regulating plant growth and plant adaption to the environment [4]–[6]. Additionally, human prefer to consume Se-rich foods because Se appears to have an critical role in strengthening the immune system in human body [7], [8]. Thus, the importance of Se for both human and plants has driven the long-term application of Se fertilizers in farm work, which is another important factor contributing to the increasing anthropogenic release of Se into the agricultural environment [9], [10].

Se with low dose often acts as a protector helping plants against various environmental stimuli [11], but the great concern has been raised about the possible adverse effects of the excessive Se in plants. Treatment with Se (8–16 ppm) significantly inhibits the growth of barley shoot [12]. Se at the concentration of 4–6 ppm show remarkable inhibitory effect on the growth of both shoot and root in bean seedlings [13]. By using image analysis of roots, the root development of lettuce and ryegrass can be completely inhibited by Se as low as 1 ppm [14]. The mechanism of Se-induced phytotoxicity is rarely reported because of the limited studies about the adverse effects of Se on plants. Several studies suggest that excessive Se can trigger oxidative stress in plants by inducing the production of reactive oxygen species (ROS) and the subsequent lipid peroxidation, which may contribute to Se-induced phytotoxicity [12], [13], [15]. A recent study indicated that Se-induced growth stunt of root was closely associated with the disturbance of plant hormones and endogenous nitric oxide (NO) in *Arabidopsis* [16], but the biochemical mechanisms for Se-induced phytotoxicity are still elusive.

Hydrogen sulfide (H_2_S), the third gasotransmitter generated endogenously in mammals after NO and carbon monoxide (CO), has been highly appreciated for its clinical relevance [17]–[20]. In plants, H_2_S is produced from cysteine desulfuration catalyzed by _L_-cysteine desulfhydrase (LCD, EC4.4.1.1) and _D_-cysteine desulfhydrase (DCD, EC4.4.1.15), both of which belonging to pyridoxal 5′-phosphate (PLP)-dependent protein family [21]. Both genes (*LCD* and *DCD*) have been characterized in *Arabidopsis* [22]–[24]. Recently, H_2_S has been drawing increasing attention in plants because it shows great potential in the regulation of multiple physiological processes in plants, but the detailed studies in the biological role of H_2_S in plants are still very limited as compared to those in mammals [25], [26]. The exogenous application of NaHS, a H_2_S donor, can alleviate the phytotoxicity induced by various metal species, such as copper (Cu) [27], chromium (Cr) [28], boron (B) [29], lead (Pd) [30], [31], aluminum (Al) [32]–[34], and cadmium (Cd) [35]–[37]. All of these reports suggest that H_2_S may be an important player regulating plant response to heavy metal stress. Nevertheless, the specific role of endogenous H_2_S in modulating the phytotoxicity induced by heavy metals (including Se) is largely unknown because of the lack of the data of tracking endogenous H_2_S *in situ* in plants. Our recent study demonstrate that Washington Stat Probe 1 (WSP-1) is a very useful fluorescent probe for selectively capturing and tracking H_2_S *in vivo* in plant root, which provides a powerful tool for identifying the role of endogenous H_2_S as a true cellular signaling molecule in regulating plant physiology [38], [39].

In this work, we investigated whether and how endogenous H_2_S responds to Se-induced toxicity in the roots of *Brassica rapa*. The effect of Se stress on the generation of endogenous H_2_S was studied *in vivo* by using fluorescent microscopy. To get deeper insights into the role of H_2_S in Se-induced toxicity, the involvement of the endogenous H_2_S in root elongation, cell death, and oxidative injury was investigated further by pharmacological experiments. These results were able to help our understanding for the role of H_2_S in plants under Se stress, which could extend our knowledge of H_2_S in plants and Se-induced phytotoxicity.

## Materials and Methods

### Plant culture and chemicals

Seeds of *B. rapa* (LvLing) seeds were surface-sterilized with 1% NaClO for 10 min followed by washing with distilled water. Seeds were germinated for 1 day in the dark on the floating plastic nets. Then the selected identical seedlings with radicles 0.5 cm were transferred to another Petri dish containing various treatment solutions in a chamber with a photosynthetic active radiation of 200 µmol/m^2^/s, a photoperiod of 12 h, and the temperature at 25±1°C.

Seedling roots were exposed to Na_2_SeO_3_ (sodium selenite, Se(IV)) with different concentrations (0.03–0.46 mM) for various treatment time (0–72 h). The 0–2.0 mM of NaHS (sodium hydrosulphide) was applied as H_2_S donor. PAG (_DL_-propargylglicine) (0.05–0.2 mM) and HT (hypotaurine) (0.1–0.4 mM) are H_2_S biosynthesis inhibitor and H_2_S scavenger, respectively. The treatment solution is composed of different chemicals as mentioned above according to the experimental design. After treatments, the roots were washed with distilled water for physiological, histochemical, and biochemical analysis.

### Histochemical analysis

The intracellular H_2_S was visualized using specific fluorescent probe WSP-1 [3′-methoxy-3-oxo-3H-spiro[isobenzofuran-1,9′-xanthen]-6′-yl 2-(pyridin-2-yldisulfanyl)benzoate] *in situ* according to our previous method [38]. The roots of seedlings after treatments were incubated at 20 mM Hepes-NaOH (pH 7.5) buffer solution containing 20 µM of WSP-1 at 25°C for 40 min. Then the roots were washed with distilled water three times and were visualized immediately by a fluorescence microscope with a 465/515 nm and an excitation/emission filter set (ECLIPSE, TE2000-S, Nikon). The relative fluorescent density of the fluorescent images was analyzed using Image-Pro Plus 6.0 (Media Cybernetics, Inc.).

Intracellular ROS was visualized using specific fluorescent probe DCFH-DA (2′,7′-dichlorofluorescein diacetate) *in situ* described by Foreman et al. [40]. The roots of seedlings were incubated in 10 µM of DCFH-DA at 25°C for 10 min. Then the roots were rinsed with distilled water for three times followed by the visualization (excitation 488 nm and emission 525 nm) with a fluorescence microscope (ECLIPSE, TE2000-S, Nikon). The relative fluorescent density of the fluorescent images was analyzed using Image-Pro Plus 6.0 (Media Cybernetics, Inc.).

Intracellular superoxide radical was visualized using specific fluorescent probe DHE (dihydroethidium) *in situ* described by Yamamoto et al [41]. The roots of seedlings after treatment were incubated in 15 µM of DHE at 25°C for 15 min. Then the roots were rinsed with distilled water for three times and were visualized (excitation 535 nm and emission 610 nm) by a fluorescence microscope (ECLIPSE, TE2000-S, Nikon). The relative fluorescent density of the fluorescent images was analysed using Image-Pro Plus 6.0 (Media Cybernetics, Inc.).

Histochemical detection of cell death was performed by using propidium iodide (PI) *in situ* as described by Kellermeier et al [42]. The roots of seedlings after treatment were incubated in 20 µM of PI solution for 20 min. Then the roots were rinsed with distilled water for three times and were visualized (excitation 535 nm and emission 615 nm) by a fluorescence microscope (ECLIPSE, TE2000-S, Nikon). The relative fluorescent density of the fluorescent images was analyzed using Image-Pro Plus 6.0 (Media Cybernetics, Inc.).

Histochemical detection of glutathione (GSH) was performed by using specific molecular probe monochlorobimane *in situ* as described by Liso et al [43]. The endogenous GSH in root was visualized after conjugation with monochlorobimane to give fluorescent GS-bimane adduct. The roots of seedlings after treatment were incubated in 100 µM of monochlorobimane solution for 30 min. Then the roots were rinsed with distilled water for three times and were visualized (excitation 390 nm and emission 478 nm) by a fluorescence microscope (ECLIPSE, TE2000-S, Nikon). The relative fluorescent density of the fluorescent images was analyzed using Image-Pro Plus 6.0 (Media Cybernetics, Inc.).

Histochemical detection of lipid peroxidation was achieved by using Schiff's regent as described by Wang and Yang [44]. The roots of seedlings after treatment were incubated in Schiff's regent for 20 min. Then the stained roots were rinsed with a solution containing 0.5% (w/v) K_2_S_2_O_5_ (prepared in 0.05 M of HCl) until the root colour became light red. After that, the roots were photographed using a digital camera.

Histochemical detection of loss of plasma membrane integrity was performed by using Evans blue as described by Yamamoto et al. [45]. The roots of seedlings after treatment were incubated in 5 ml of 0.025% Evans blue solutions (w/v) for 20 min. After that, the roots were rinsed with distilled water for three times followed by photographed using a digital camera.

### Screening and analysis of *LCD* and *DCD* from the genome of *B. rapa*

The sequences of *AtLCD* (AT5G28030) and *AtDCDs* (AT3G26115 and AT2G48420) from *Arabidopsis* were used as baits for BLAST research in the genome of *B. rapa* from BRAD (http://brassicadb.org/brad/index.php). The obtained sequences were retrieved and analyzed. The phylogenetic trees were constructed using the maximum likelihood method in MEGA 5.2. The multialignment of amino acid sequences was performed with ClustalX 2.0 and DNAMAN 5.2.2. Protein structure prediction was performed on SMART (http://smart.embl-heidelberg.de/).

The DNA sequences with the length of 2 kb were retrieved from the upstream of *LCDs* and *DCDs* in *B. rapa* for promoter analysis. The sequence between the start of target gene and the end of its upstream gene was obtained for promoter analysis if the length of this sequence was less than 2 kb. The *cis*-elements in the retrieved promoter regions were analyzed using online tool PLACE (http://www.dna.affrc.go.jp/PLACE/signalscan.html).

### Analysis of transcripts

Total RNA was extracted from root tissues using Trizol (Invitrogen) according to the manufacturer's instructions. Reverse transcription was performed at 42°C in 25 µl reaction mixture including 3 µg of RNA, 0.5 µg of oligo (dT) primers, 12.5 nmol of dNTPs, 20 units of RANase inhibitor and 200 units of M-MLV. The first cDNA was used as a template for polymerase chain amplification and to analyse the transcripts of genes by using real-time quantitative reverse transcription-polymerase chain reaction (qRT-PCR) (Applied Biosystems 7500 Fast Real-Time PCR System, LifeTechnologies). The primers designed for the amplification of the genes are listed in Table S1.

### Measurement of Se concentration in roots

About 0.2 g of dried root sample was mixed with 10 mL of HNO_3_∶HClO_4_ (4∶1, v/v) in a test tube and covered with Parafilm for 24 h. Then the samples were digested. The digested solution was analyzed for Se concentration by using hydride generation atomic fluorescence spectrometry (AFS-230a, Beijing Wantuo). The calibration was performed by using standard Se solution with concentration of 10–80 µg/L [9].

### Statistical analysis

Each result was presented as the mean ± standard deviation (SD) of at least three replicated measurements. The significant differences between treatments were statistically evaluated by SD and one-way analysis of variance (ANOVA) using SPSS 2.0. The data between two specific different treatments were compared statistically by ANOVA, followed by F-test if the ANOVA result is significant at *P*<0.05. For multiple comparison analysis, least significant difference test (LSD) was performed on all data following ANOVA tests to test for significant (*P*<0.05) differences among different treatments.

## Results

### Se(IV) treatment inhibited root elongation of *B. rapa*

**Figure 1:**
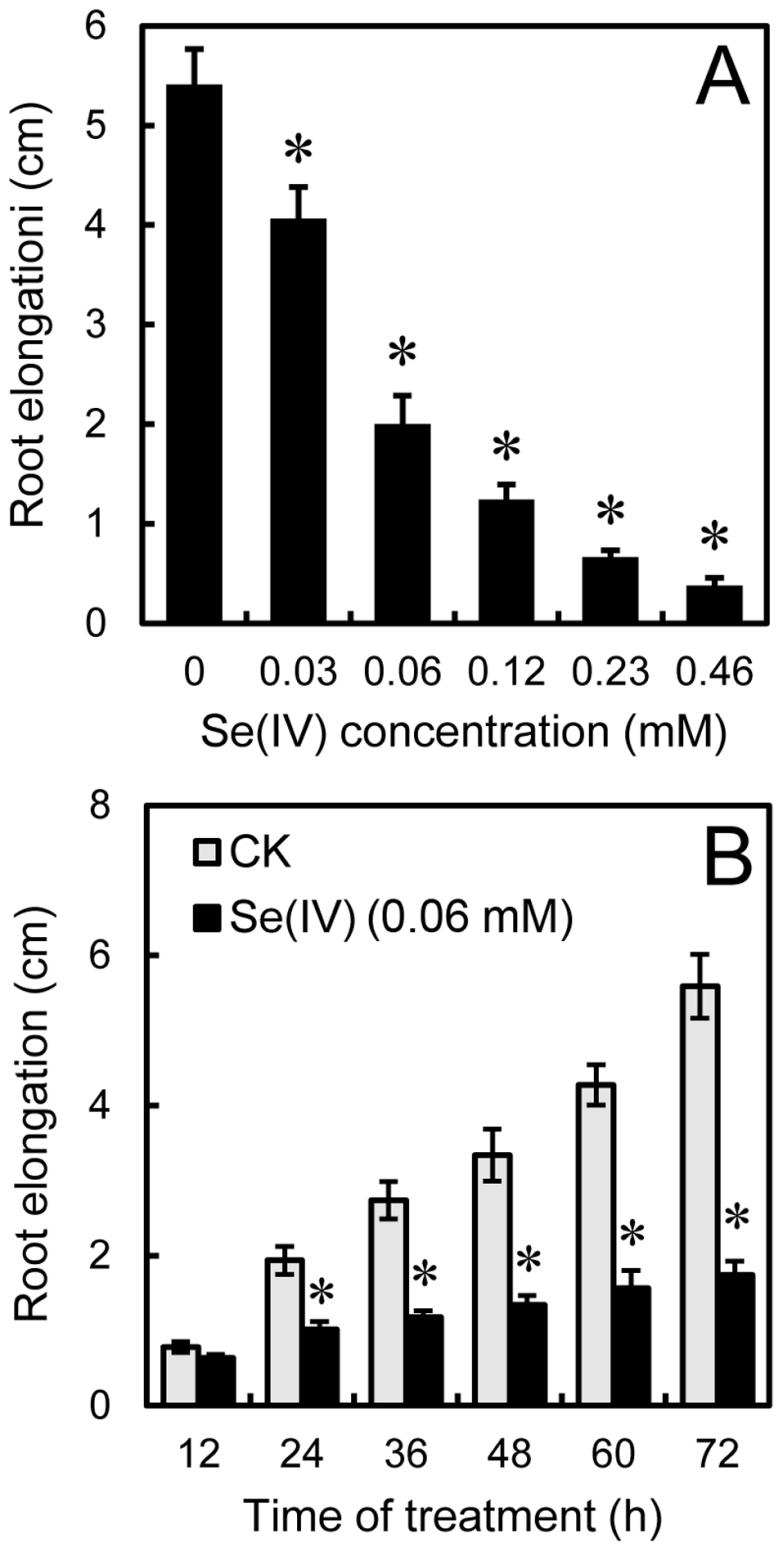
Effect of Se(IV) on the root growth of *B. rapa*. (A) The roots of seedlings were exposed to 0, 0.03, 0.06, 0.12, 0.23, and 0.46 mM of Se(IV) solution for 72 h. After that, the root length were measured. (B) The root length were obtained when the roots of seedlings were exposed to 0.06 mM of Se(IV) solution for 12, 24, 36, 48, 60, and 72 h, respectively. *Asterisk* indicates that mean values of three replicates are significantly different between the treatments of Se(IV) and the control group (CK) (*P*<0.05).

Treatment with Se(IV) significantly inhibited root elongation in both dose- and time-dependent manners. The roots of *B. rapa* were exposed to 0–0.46 mM of Se(IV) for up to 72 h. Compared to the control group, root elongation decreased by 25%, 63%, 77%, 88%, and 93% at 0.03, 0.06, 0.12, 0.23, and 0.46 mM of Se(IV) levels, respectively (Figure 1A). In a time-course experiment, exposure of 0.06 mM of Se(IV) showed significantly inhibitory effect on root elongation. Compared to the control group, root elongation began to decreased after treatment with 0.06 mM of Se(IV) for 24 h, and continued to decrease up to 72 h (Figure 1B).

### Se(IV) treatment inhibited the generation of endogenous H_2_S in root tips

**Figure 2:**
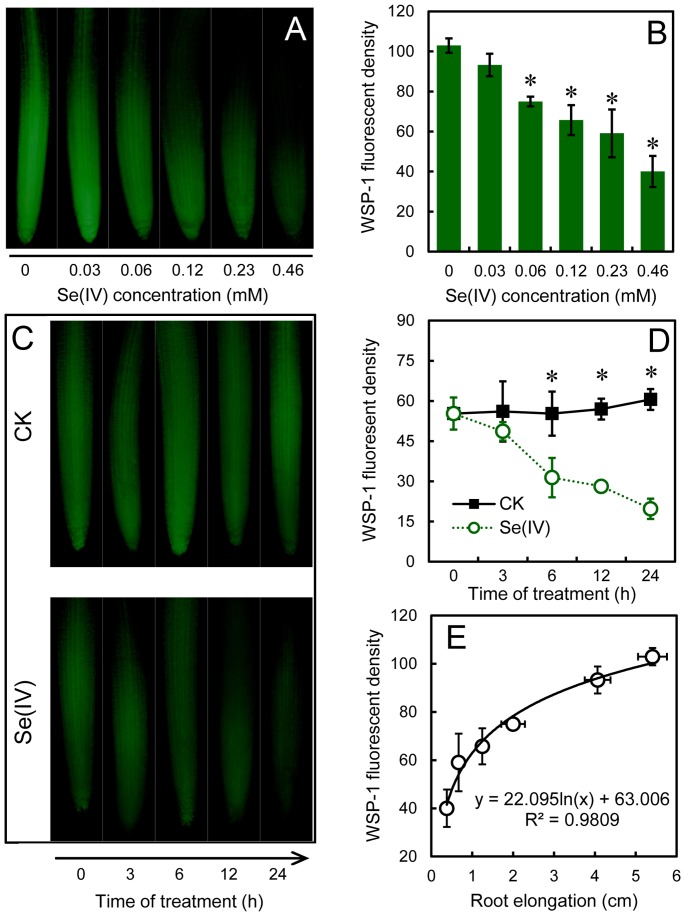
Effect of Se(IV) on the endogenous H_2_S in the root tips of *B. rapa*. The roots of seedlings were exposed to 0, 0.03, 0.06, 0.12, 0.23, and 0.46 mM of Se(IV) solution for 48 h. Afterwards, the roots were loaded with WSP-1 for fluorescent imaging (A) and the calculation of relative fluorescent density (B). (C–D) The image and density of WSP-1 fluorescence were obtained when the roots of seedlings were exposed to 0.06 mM of Se(IV) solution for 0, 3, 6, 12, and 24 h, respectively. (E) The correlation analysis between WSP-1 fluorescent density and root elongation under Se(IV) treatment with concentration at 0, 0.03, 0.06, 0.12, 0.23, and 0.46 mM. *Asterisk* indicates that mean values of three replicates are significantly different between the treatments of Se(IV) and the control group (CK) (*P*<0.05).

Root tip is the main expansion zone for root elongation [46]. In order to test the effect of Se(IV) stress on endogenous H_2_S in root tips, we performed *in situ* detection of endogenous H_2_S generation by using specific fluorescent probe WSP-1. Compared to the control, the decreased WSP-1 fluorescent density was observed in root tips in the presence of Se(IV) in a dose-dependent manner (Figure 2A and B). In a time-course experiment, the endogenous H_2_S indicated by WSP-1 fluorescence maintained stable up to 24 h in control groups. However, WSP-1 fluorescence began to decrease significantly after treatment with 0.06 mM of Se(IV) for 6 h (Figure 2C and D). The correlation analysis suggested that the changes of endogenous H_2_S level occurred in parallel with the changes of root elongation under Se(IV) stress. Initially, WSP-1 fluorescent density decreased slowly with the light decrease in root elongation, followed by a quick decrease with the dramatic inhibition of root elongation induced by Se(IV) at high concentrations (Figure 2E). These results suggested that the generation of endogenous H_2_S decreased significantly in root tips upon Se(IV) treatment.

### Se(IV) stress differentially regulated the expression of *LCD* and *DCD* in roots

In order to understand how Se(IV) stress impacted the generation of endogenous H_2_S, we further investigated the effect of Se(IV) stress on the expression of *LCD* and *DCD* in the roots of *B. rapa*. According to BLAST search, sequence identity, and phylogenetic analysis, we obtained two *DCD* homologues (Bra025184 and Bra018726) and ten *LCD* homologues (Bra020605, Bra001131, Bra014529, Bra004781, Bra006115, Bra037682, Bra039708, Bra009985, Bra036910, and Bra006114) from *B. rapa* (Figure S1). All of the retrieved LCDs and DCDs have typical PLP domains (Figure S2). *LCD* has been well studied in *Arabidopsis* and *Brassica napus* [23], [47]. The multialignment of deduced amino acid sequences revealed that the obtained LCDs from *B. rapa* had many typically structural features of plant LCDs, such as PLP-binding sites, the substrate binding site, and the SAT protein-interaction site (Figure S3) [47].

**Figure 3:**
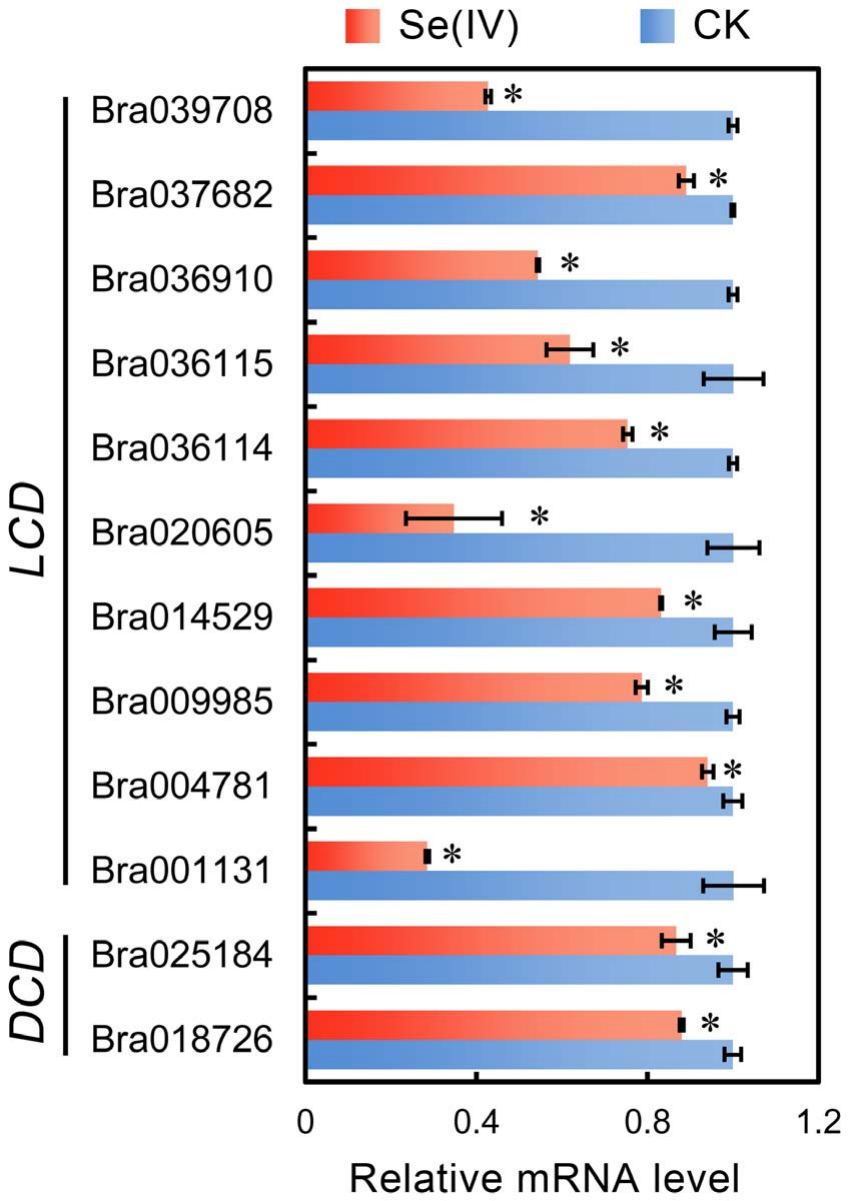
Effect of Se(IV) on the expression of *LCDs* and *DCDs* in the roots of *B. rapa*. The roots of seedlings were exposed to 0.06 mM of Se(IV) solution for 48 h. The total RNA was extracted from roots for qRT-PCR analysis. *Actin* was used for cDNA normalization. *Asterisk* indicates that mean values of three replicates are significantly different between the treatments of Se(IV) and the control group (CK) (*P*<0.05).

The expression levels of *LCDs* and *DCDs* under 0.06 mM of Se(IV) treatment were tested by using qRT-PCR (Figure 3). The results suggested that Se(IV) stress showed extensively inhibitory effect on the expression of both *LCDs* and *DCDs* (Figure 3). Two *DCDs* in roots were down-regulated upon Se(IV) stress (Figure 3). Compared to the control, Se(IV) treatment decreased the expression of most *LCDs*. Among them, Bra001131, Bra020605, and Bra039708 showed relatively more decreased transcription as compared to their controls, respectively (Figure 3). These results suggested that Se(IV)-induced inhibition of endogenous H_2_S might resulted from the down-regulation of *LCDs* and *DCDs* in the roots of *B. rapa*.

### Analysis of nitric oxide-, auxin-, and metal-responsive *cis*-elements in the promoter region of *LCDs* and *DCDs* in roots

According to the identification of NO-responsive *cis*-element (NRE) from higher plants [48], [49], several NREs (e.g. ACGT Box, MYCL, and W-BOX) could be found in the promoter region of all the *LCDs* and *DCDs* obtained from *B. rapa* (Table S2). In addition, the auxin-responsive *cis*-element (ARE) could be found in most *LCDs* and *DCDs* except for Bra018726 (Table S2).

### Application of H_2_S donor NaHS alleviated Se(IV)-induced root inhibition by enhancing endogenous H_2_S level

**Figure 4:**
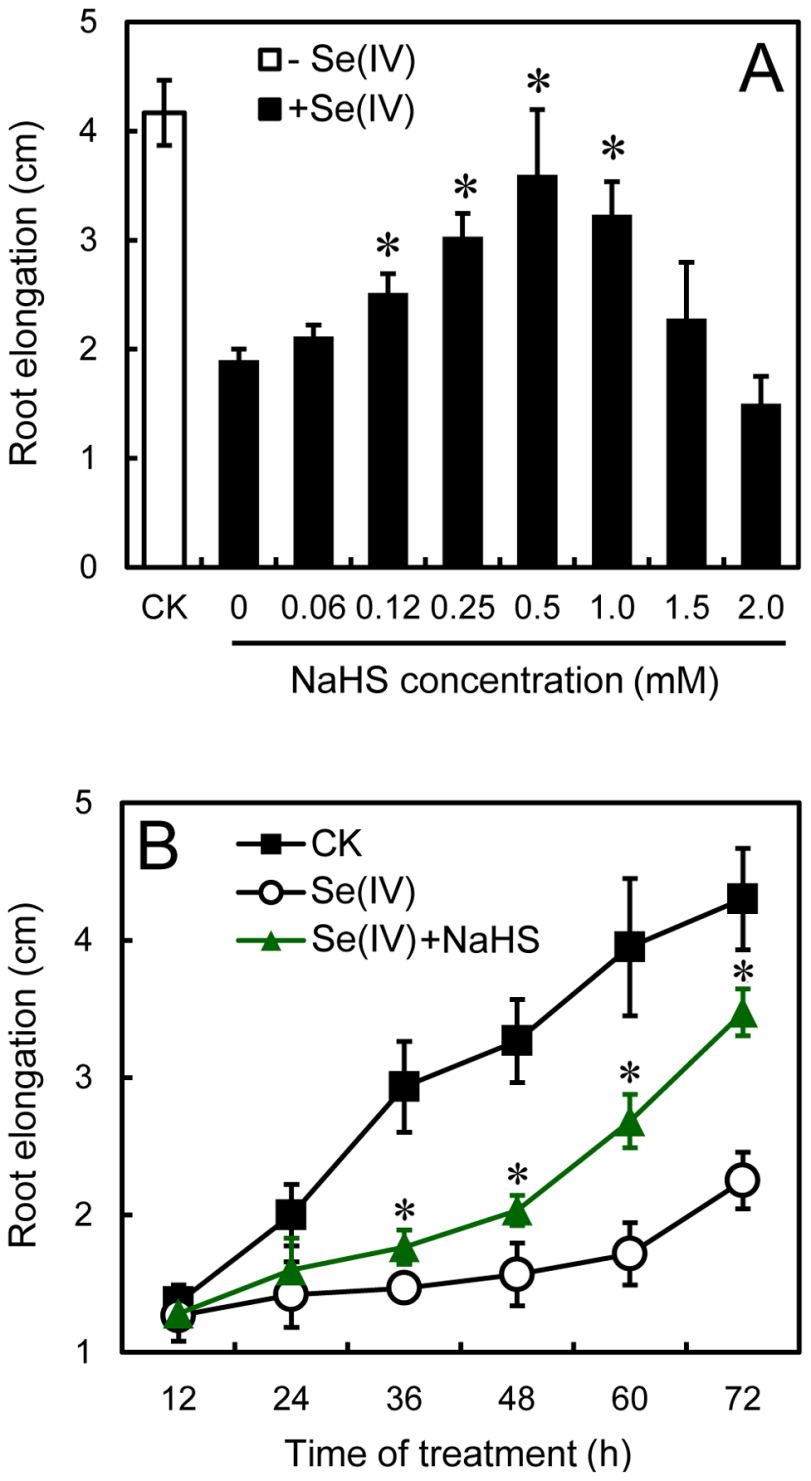
Effect of NaHS on the root elongation under Se(IV) stress. (A) In the presence of Se(IV) at 0.06 mM, the roots were treated with NaHS with different concentrations (0–2.0 mM) for 72 h. After that, the root elongation was measured. *Asterisk* indicates that mean values of three replicates are significantly different between the treatments of Se(IV) and Se(IV)+NaHS (CK) (*P*<0.05). (B) The roots were exposed to 0.06 mM of Se(IV) and 0.5 mM of NaHS simultaneously for 12, 24, 36, 48, 60, and 72 h. After that, the root elongation was measured. *Asterisk* indicates that mean values of three replicates are significantly different between the treatments of Se(IV) and the control group (CK) (*P*<0.05).

To obtain more evidence for the involvement of H_2_S in the regulation of root elongation under Se(IV) stress, the H_2_S donor NaHS was added to the treatment solution. A preliminary experiment with NaHS at 0.06–2.0 mM was carried out to determine the point where NaHS showed the most significant effect. Treatment with NaHS at 0.5 mM had the greatest effect on the alleviation of Se(IV)-induced inhibition of root elongation (Figure 4A). The root elongation increased by 90% in seedlings treated with 0.5 mM NaHS+0.06 mM Se(IV) as compared to 0.06 mM Se(IV) treatment alone (Figure 4A). In a time-course experiment, Se(IV)-induced reduction in root elongation was significantly recovered when roots were incubated in the treatment solution containing both Se(IV) and 0.5 mM of NaHS (Figure 4B), which may resulted from the enhancement of endogenous H_2_S level.

**Figure 5:**
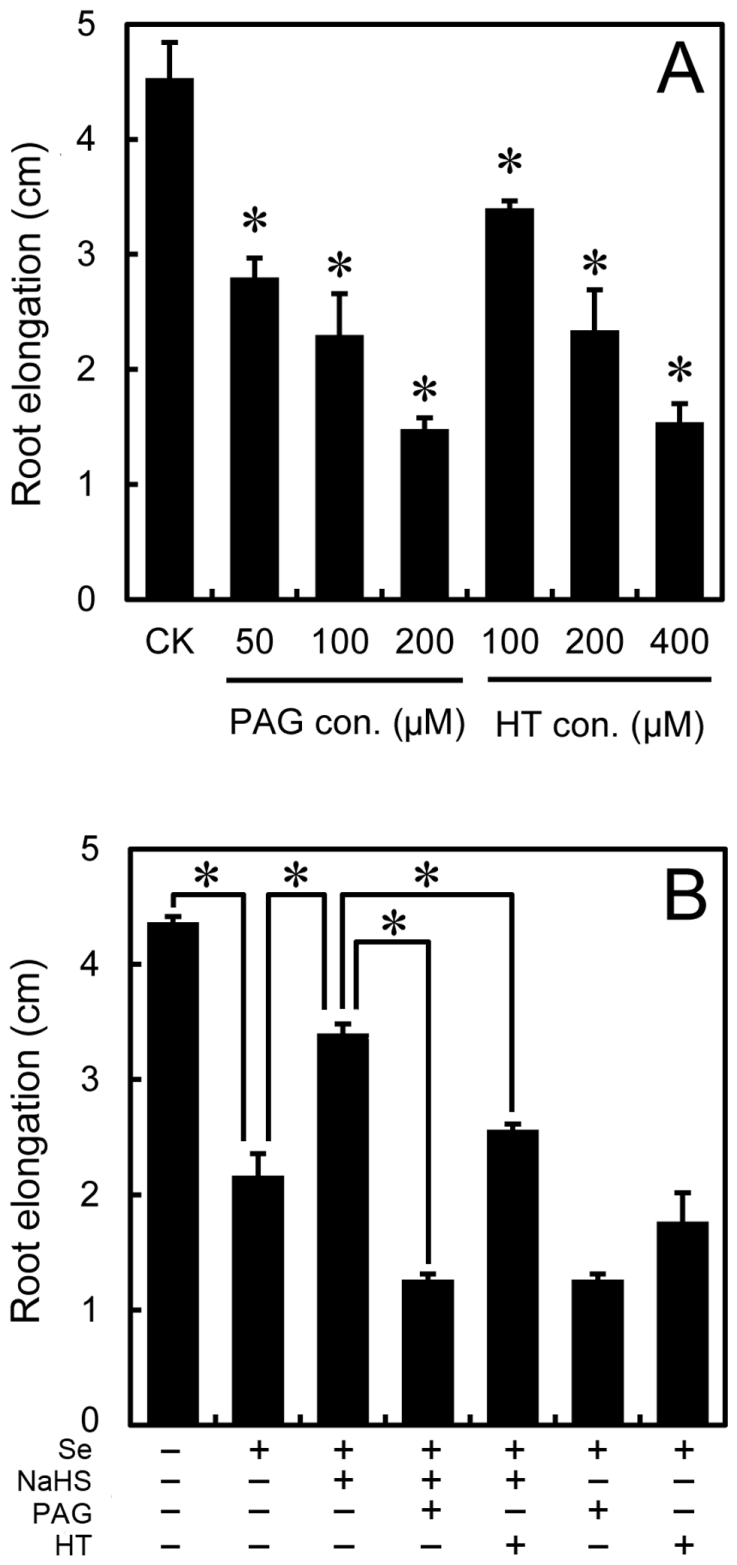
Effect of PAG and HT on root elongation under Se(IV) stress. (A) The roots were exposed to PAG (50, 100, and 200 µM) or HT (100, 200, 400 µM) for 72 h. After that, the root elongation was measured. *Asterisk* indicates that mean values of three replicates are significantly different between the treatments of Se(IV) and the control group (CK) (*P*<0.05). (B) The roots were treated with water, 0.5 mM of NaHS, 100 µM of PAG, and 200 µM of HT alone or their combinations. After various treatments for 72 h, the root elongation was measured. *Asterisk* indicates that mean values of three replicates are significantly different between different treatments indicated.

To verify the alleviated effect of NaHS on root elongation under Se(IV), we tested the root elongation treated with PAG (endogenous H_2_S biosynthesis inhibitor) and HT (H_2_S scavenger), respectively. Compared to the control, PAG and HT resulted in the significant decreases in root elongation (Figure 5A), respectively, suggesting that the endogenous H_2_S is essential for root elongation. Furthermore, the addition of PAG or HT could partially blocked the alleviated effect of NaHS on Se(IV)-induced root inhibition (Figure 5B), which may resulted from the decrease in endogenous H_2_S level.

**Figure 6:**
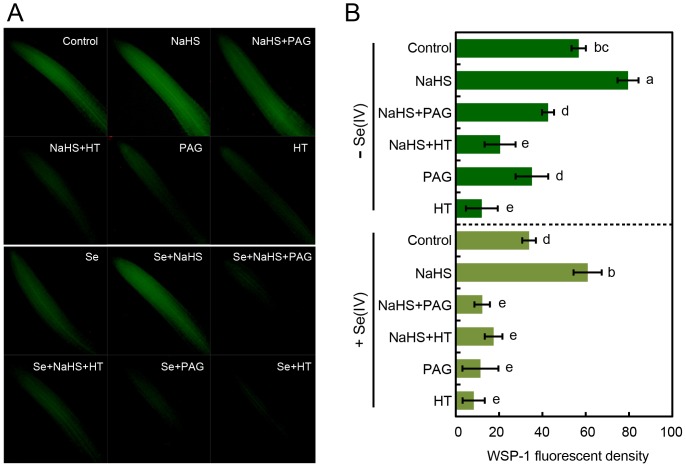
Effect of NaHS, PAG, and HT on the endogenous H_2_S level in root tips with or without Se(IV) stress. In the presence of 0.06 mM of Se(IV) or not, the roots were treated with water, 0.5 mM of NaHS, 100 µM of PAG, and 200 µM of HT alone or their combinations. After various treatments for 3 h, the roots were loaded with WSP-1 for fluorescent imaging (A) and the calculation of relative fluorescent density (B). The mean values of three replicates followed by different letters indicate significance of difference between the treatments (*P*<0.05, ANOVA, LSD).

Subsequently, we test the effect of NaHS application on the endogenous H_2_S level in roots under Se(IV) stress. In Se(IV)-free roots, NaHS could enhance the level of endogenous H_2_S while both PAG and HT were able to decrease endogenous H_2_S level (Figure 6). Additionally, the addition of NaHS could recover the decrease in endogenous H_2_S level in Se(IV)-treated roots. However, in both Se(IV)-free and Se(IV)-treated roots, the enhancement of endogenous H_2_S level by NaHS supplement could be blocked by the addition of PAG and HT, respectively (Figure 6). All of these results suggested that the enhancement of endogenous H_2_S could alleviate Se(IV)-induced inhibition in root elongation.

### Treatment with NaHS attenuated Se(IV)-induced ROS generation, cell death, and oxidative injury in roots

**Figure 7:**
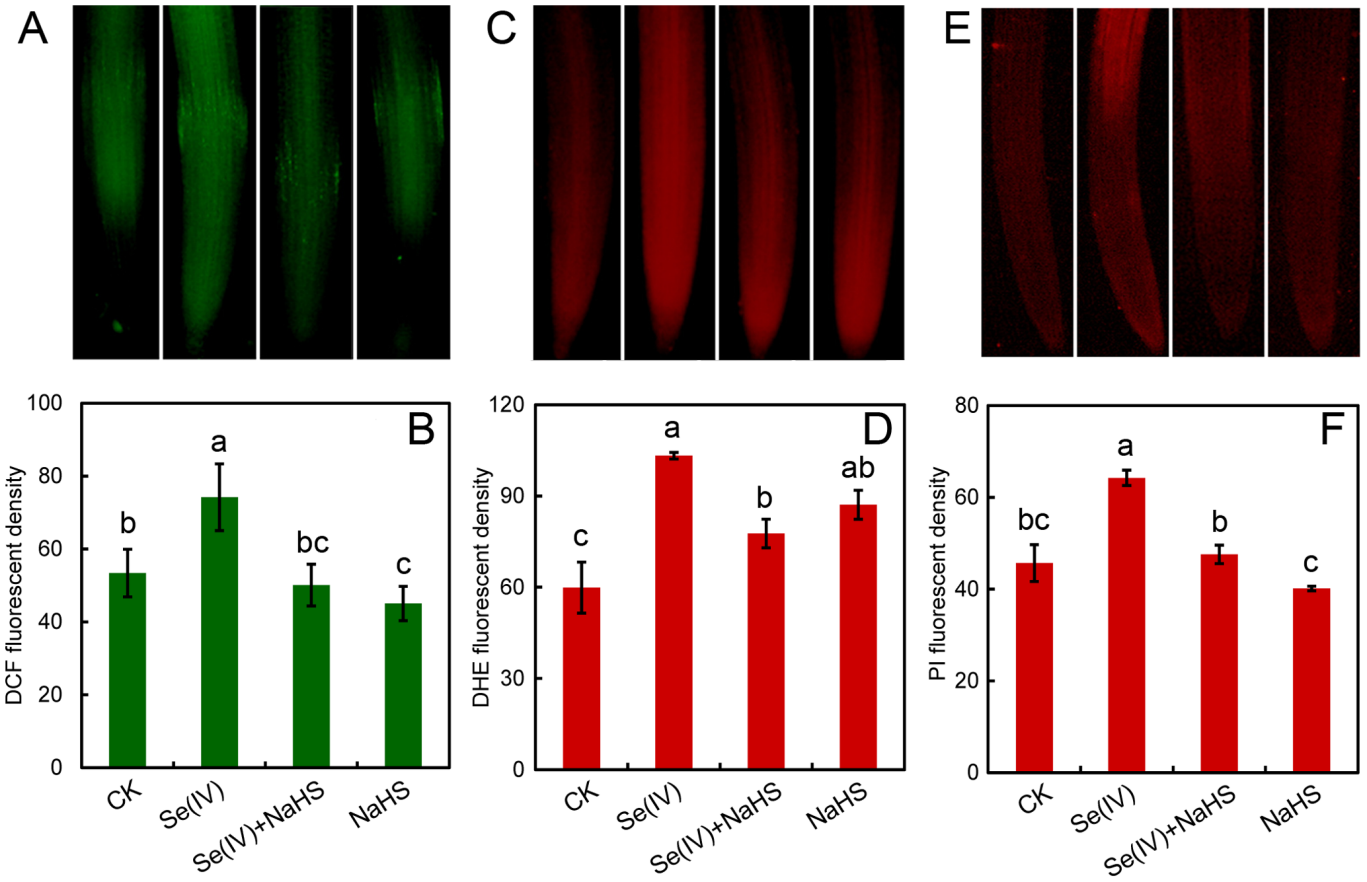
Effect of NaHS on endogenous ROS, super oxide radical, and cell death in root tips under Se(IV) stress. The roots were exposed to water, 0.06 mM of Se(IV), 0.06 mM of Se(IV) and 0.5 mM of NaHS, and 0.5 mM of NaHS for 3 h. Then roots were loaded with DCFH-DA (A), DHE (C), and PI (E) for fluorescent imaging, respectively. The fluorescent density of DCF (B), DHE (D), and PI (F) was estimated, respectively. The mean values of three replicates followed by different letters indicate significance of difference between the treatments (*P*<0.05, ANOVA, LSD).

Compared to the control, treatment with 0.06 mM of Se(IV) resulted in the over-generation of total endogenous ROS in root tips indicated by staining with specific fluorescent probe DCFH-DA. However, the addition of NaHS significantly decreased the accumulation of total ROS induced by Se(IV) (Figure 7A and B). Superoxide radical, one of the most important ROS, was detected with specific fluorescent probe DHE. The addition of NaHS significantly inhibited the increase in superoxide radical level in root tips under Se(IV) stress (Figure 7C and D).

**Figure 8:**
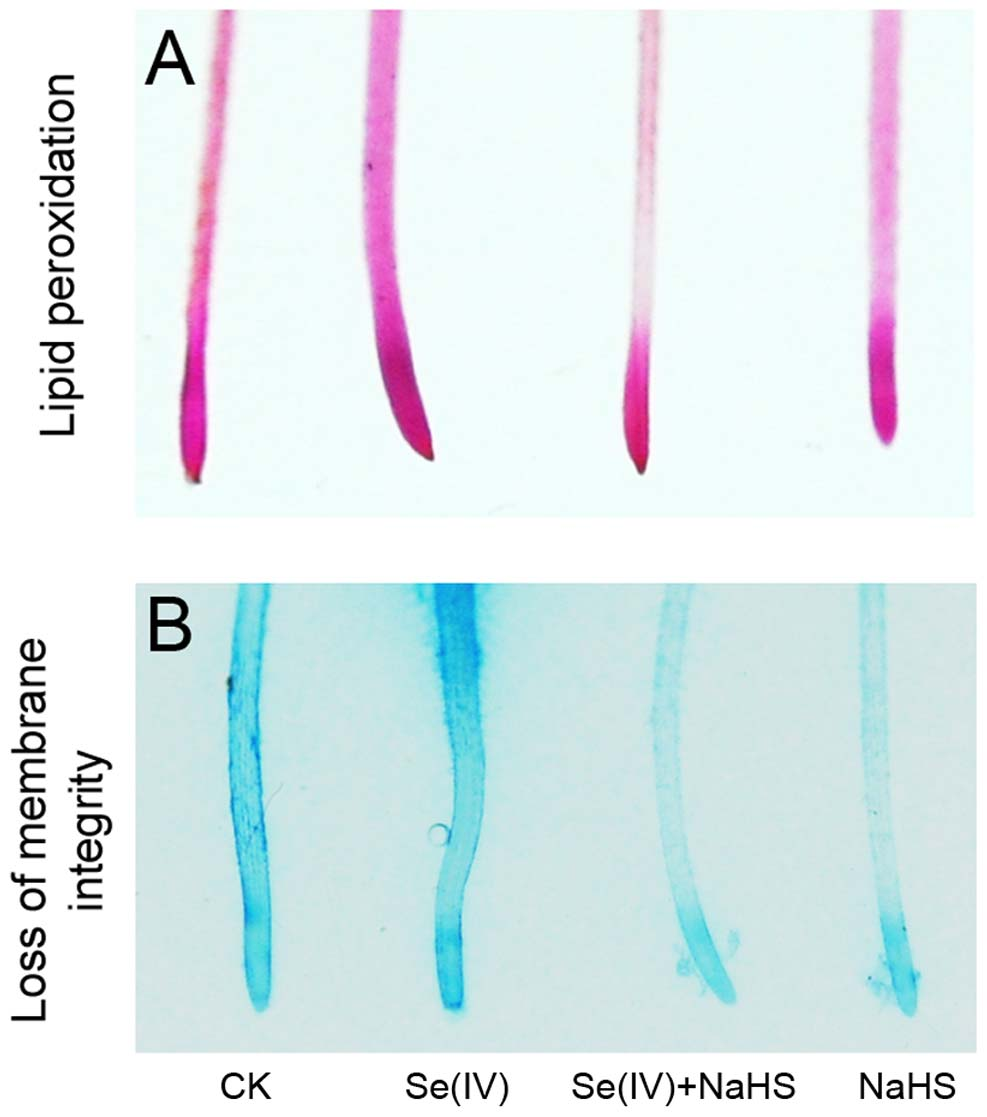
Effect of NaHS on lipid peroxidation and the loss of plasma membrane integrity in roots under Se(IV) stress. The roots were exposed to water, 0.06 mM of Se(IV), 0.06 mM of Se(IV) and 0.5 mM of NaHS, and 0.5 mM of NaHS for 48 h. Then the roots were stained with Schiff's reagent (A), and Evan blue (B) for imaging, respectively.

Cell death in root tips were fluorescently detected with PI. The application of NaHS was able to significantly alleviate Se(IV)-induced cell death in root tips (Figure 7E and F). Because the over-generation of ROS is closely related to the oxidative injury to plant cells, we further determined the peroxidation of membrane lipids and the loss of membrane integrity by using histochemical staining with Shiff's regent and Evans blue, respectively [44]. Compared to the control, the roots treated with Se(IV) showed more extensive staining. However, the roots treated with Se(IV)+NaHS had only light staining as compared to Se(IV) treatment alone (Figure 8). These results indicated that the enhancement of endogenous H_2_S by applying NaHS could alleviate Se(IV)-induced cell injury in root tips.

### Treatment with NaHS enhanced endogenous GSH level in Se(IV)-treated roots

**Figure 9:**
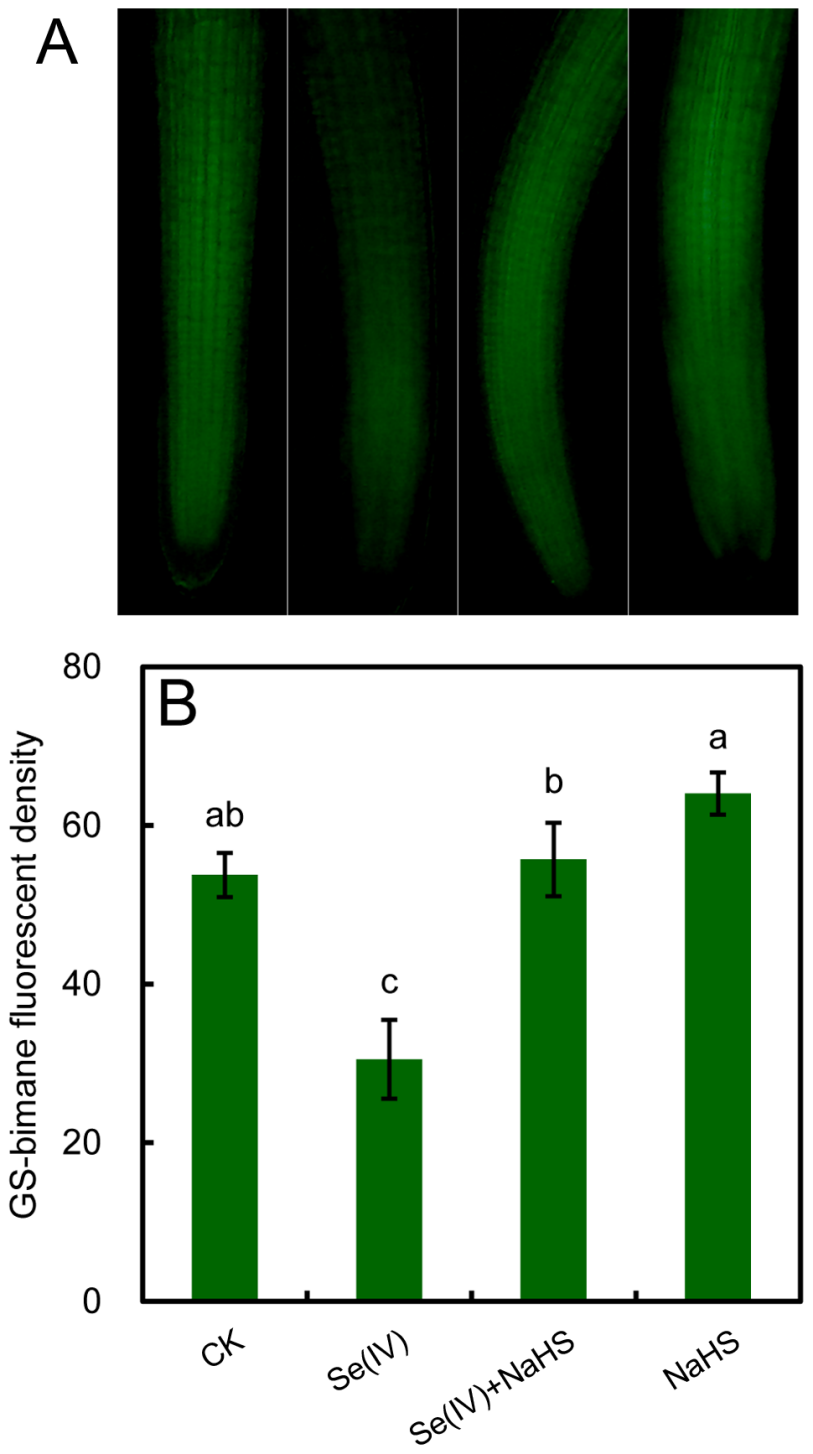
Effect of NaHS on endogenous GSH in roots under Se(IV) stress. The roots were exposed to water, 0.06 mM of Se(IV), 0.06 mM of Se(IV) and 0.5 mM of NaHS, and 0.5 mM of NaHS for 48 h. Afterwards, the roots were loaded with WSP-1 for fluorescent imaging (A) and the calculation of relative fluorescent density (B). The mean values of three replicates followed by different letters indicate significance of difference between the treatments (*P*<0.05, ANOVA, LSD).

By using specific molecular probe for detecting endogenous GSH *in situ*, treatment with 0.06 mM of Se(IV) significantly decreased the endogenous GSH level in root as compared to the control. However, the addition of NaHS could remarkabley enhance the GSH level (Figure 9A). The relative GS-bimane fluorescent density indicated that treatment with Se(IV)+NaHS increased the endogenous GSH by 82.6% as compared to Se(IV) treatment alone (Figure 9B).

### The concentration of Se in roots were not affected effectively by the application of NaHS

In order to test the effect of NaHS treatment on the uptake of Se by the roots, the Se concentration in roots were measured and compared between Se(IV) treatment and Se(IV)+NaHS treatment. In a time-course experiment, treatment with 0.6 mM of Se(IV) resulted in the continuous increase in concentration of Se in roots as compared to the control groups (Figure S4). However, the addition of 0.5 mM of NaHS didn't affect the concentration of Se in roots significantly as compared to the treatment of Se(IV) alone (Figure S4).

## Discussion

In comparison with other heavy metals (e.g. Cd, Zn, Al, Pb, and Hg), whose phytotoxicity have been well documented [50]–[53], the biochemical mechanisms for plant responses to Se are rarely known. It has been demonstrated that the exogenous application of H_2_S can modify plant physiology in response to heavy metal stress [25]. However, whether and how the endogenous H_2_S influences plant growth under heavy metal stress remains unclear. In the present study, by using *in situ* fluorescent tracking of endogenous H_2_S, we found that the inhibition of endogenous H_2_S generation underlay Se(IV)-induced inhibition of root elongation in *B. rapa*, which could be supported by four lines of evidence. First, Se(IV)-induced inhibition of root elongation was closely correlated with the decrease in endogenous H_2_S in root tips. Second, Se(IV)-induced inhibition of endogenous H_2_S generation may result from the down-regulation of *LCDs* and *DCDs*. Third, the application of H_2_S donor NaHS could enhance endogenous H_2_S level in root tips, which further resulted in the recovery of root elongation under Se(IV) stress. The decrease in endogenous H_2_S level by the addition of PAG and HT could block the recoverable effect of NaHS on root elongation under Se(IV) stress. Fourth, the enhancement of endogenous H_2_S by NaHS resulted in the alleviation of Se(IV)-induced ROS accumulation, cell death, and oxidative injury in root tips.

It has been reported that H_2_S is required for the organogenesis of lateral root and adventitious root [38], [54]–[56]. In the present study, decreasing the endogenous H_2_S level using PAG or HT could inhibit the root elongation of *B. rapa* (Figure 5A, suggesting that endogenous H_2_S is indispensable for root elongation. Se(IV)-induced inhibition of endogenous H_2_S generation in root tips may probably contributed to the depression of root elongation (Figure 1 and 2). Several *LCDs* and *DCDs* responsible for the endogenous generation of H_2_S have been identified from plants [23], [24], [47], but the regulation of the expression of these genes by environmental stimuli is rarely reported. In this study, we detected the genome-wide expression pattern of *LCDs* and *DCDs* in *B. rapa* under Se(IV) stress. We found an effective inhibitory action of Se(IV) to the expression of *LCDs* and *DCDs* in the roots of *B. rapa* (Figure 3), which may contributed to the significant decrease in endogenous H_2_S level (Figure 2). The similarly depressed mode of *LCD* expression was also observed in *B. napus* under Cd stress [47]. Interestingly, we also found some typical NO- or auxin-responsive elements in the promoter region of *LCDs* (Table S2), which may explain the previous observation that exogenous NO and auxin could stimulate the expression of *LCD* in *B. napus* [47]. Supported by genetic evidences it was proposed that the inhibition of endogenous NO and auxin contributed to Se(IV)-induced root growth inhibition in *Arabidopsis* [16]. Thus, it can an indication that NO and auxin may act upstream of H_2_S by manipulating the expression of *LCDs* and *DCDs* during Se(IV)-induced inhibition of root growth. In addition, the transcriptional regulation of *LCDs* and *DCDs* in *B. rapa* by Se(IV) may also results from the presence of MREs in their promoter region.

ROS has been suggested as the main inducer of plant cell death [57]. In the present study, Se(IV)-induced over-accumulation of ROS may contribute to the cell death in root tips, which was also accompanied with the decrease in endogenous H_2_S (Figure 2 and 7). The enhancement of endogenous H_2_S by supplement with NaHS could reverse the inducible effect of Se(IV) on ROS accumulation, cell death, and oxidative injury (Figure 7), suggesting that H_2_S has an important role in the plant protection from Se(IV) stress by scavenging the over-accumulated of ROS. In mammals, the effective stimulation of GSH biosynthesis induced by low level of H_2_S contribute to the suppression of oxidative stress more efficiently than the scavenging of ROS by H_2_S itself [58]. Cystine is indispensable for the biosynthesis of glutathione. H_2_S can enhance the activity of cysteine/glutamate antiporter, leading to the increase in the transport of cystine into cells [59]. Cystine is subsequently reduced to cysteine in cells and incorporated into glutathione. Additionally, H_2_S can directly interact with glutamylcysteine synthase, a limiting enzyme for glutathione biosynthesis, thereby increasing the production of glutathione [59], [60]. Our present results demonstrated that the increase in endogenous H_2_S by applying NaHS significantly enhance GSH level in roots under Se(IV) stress (Figure 9). Glutathione plays important role in protecting plants from metal toxicity by scavenging ROS or chelating metals [61]. Plants share similar mechanism with mammals for glutathione biosynthesis [62]. Therefore, in Se(IV)-treated plants, whether H_2_S depressed the generation of ROS through the similar mechanism mentioned above remains to be elucidated.

The biology of H_2_S in mammals has been significantly advanced, but evaluating the role of endogenous H_2_S in plants is just beginning. By using *in situ* fluorescent detection of endogenous H_2_S in plant, we provide the direct evidence that Se(IV) stress can inhibits the generation of endogenous H_2_S in the roots of *B. rapa*. The enhancement of endogenous H_2_S can alleviate Se(IV)-induced root inhibition by depressing ROS generation and decreasing cell death. These data support the fact that Se(IV) induces phytotoxicity by hijacking the generation of endogenous H_2_S in *B. rapa*. Despite of the observation in this study, H_2_S-mediated signaling components upon Se stress is still elusive. Thus, a more precise understanding of this question will accelerate the investigation on the mechanism of Se-induced phytotoxicity, which in turn will help the improvement of crop production in Se-polluted environment.

## Supporting Information

Figure S1 **The phylogenetic relationship of LCDs and DCDs in** ***B. rapa*** **with their related member in higher plants.** NCBI accession numbers are NP_974843.1 for *Arabidopsis thaliana* LCD (AtLCD), AFS17242.1 for *Brassica napus* LCD (BnLCD), NP_175275.3 for *Arabidopsis thaliana* DCD1 (AtDCD1), NP_974363.1 for *Arabidopsis thaliana* DCD2 (AtDCD2), NP_001234368.1 for *Solanum lycopersicum* DCD (SlDCD), XP_007037066.1 for *Theobroma cacao* DCD1 (TcDCD1), XP_007037067.1 for *Theobroma cacao* DCD2 (TcDCD2), XP_007037068.1 for *Theobroma cacao* DCD3 (TcDCD3), and XP_003631148 for *Medicago truncatula* DCD (MtDCD).
(TIF)

Figure S2 **The location of PLP-dependent domain in LCDs and DCDs from** ***B. rapa*** **.** The protein structure of two LCDs and two DCDs were analyzed by online tool SMART. The typical PLP-dependent domains were indicated as orange box. Bar indicated 100 amino acids (aa).
(TIF)

Figure S3 **Alignment of the predicted amino acid sequences of LCDs in** ***A. thaliana*** **,** ***B. napus*** **, and** ***B. rapa*** **.** *Dark shading* with *white letters* and *gray shading* with black letters reveal 100% and 75% sequence similarity, respectively. Database accession numbers are the same as described in Figure S1. The PLP-binding sites are shown by *red box*. The substrate binding site is indicated by *blue box*. The SAT protein-interaction site is indicated by *red box*.
(TIF)

Figure S4 **The concentration of Se in the roots of** ***B. rapa*** **exposed to Se(IV) or Se(IV)+NaHS.** The roots were exposed to Se(IV) (0.06 mM) or Se(IV) (0.06 mM)+NaHS (0.5 mM) for 6, 12, 24, 48, 72 h, respectively. The roots were harvested at each point of treatment time for Se analysis. Each value was presented as the mean of three replicates with SD.
(TIF)

Table S1 **Sequences of oligonucleotide primers for qRT-PCR.** F: forward; R: reverse.
(DOCX)

Table S2 **Distribution of** ***cis*** **-elements response to NO (NRE) and auxin (ARE) in the promote region of** ***LCDs*** **and** ***DCDs*** **in** ***B. rapa*** **.**
(DOCX)
