# Supplementary figures and images for: Selenium Inhibits Root Elongation by Repressing the Generation of Endogenous Hydrogen Sulfide in Brassica rapa
Source: PLoS One. 2014 Oct 21;9(10):e110904. doi: 10.1371/journal.pone.0110904 (PMC4204939; doi:10.1371/journal.pone.0110904)

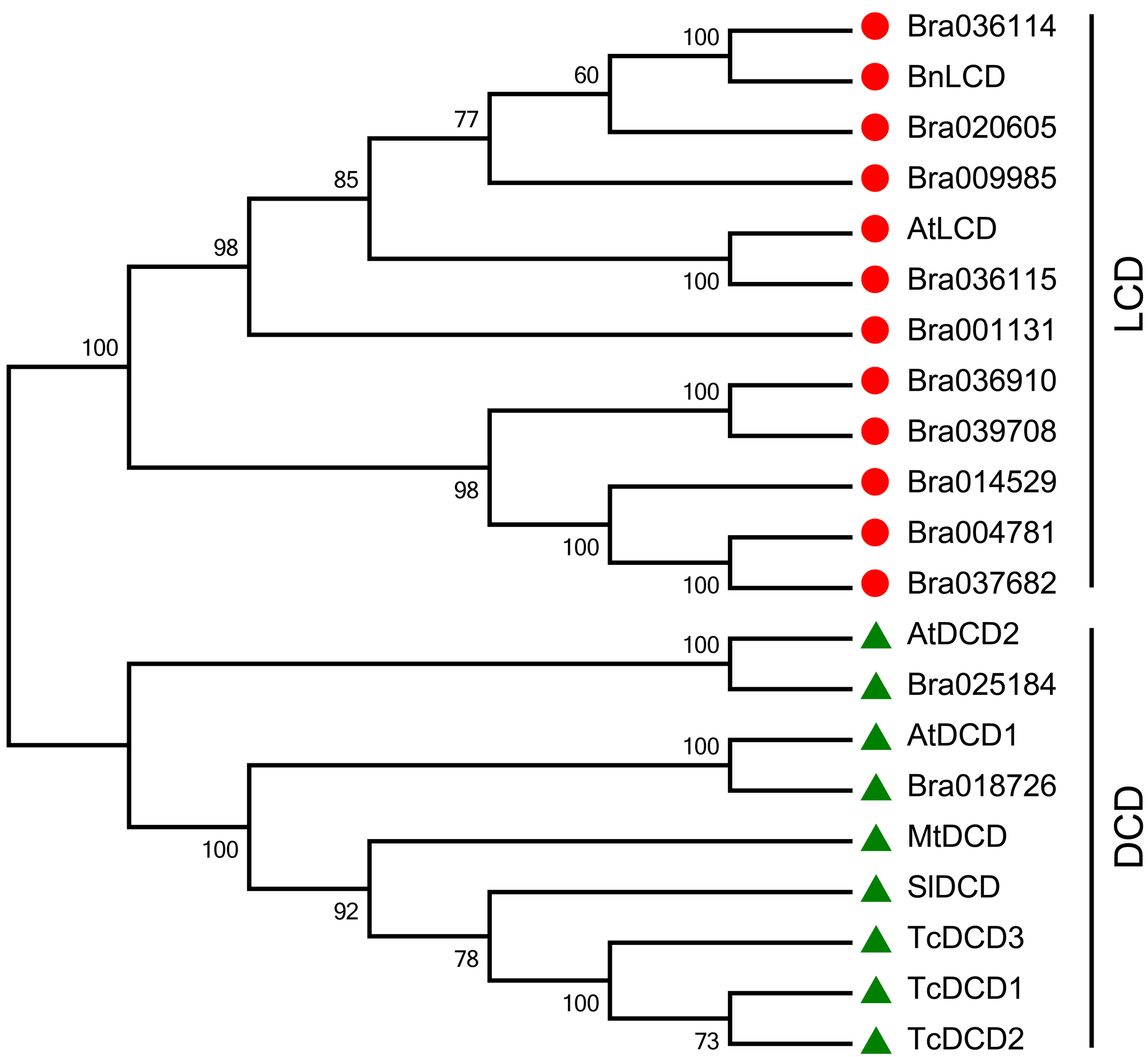

Supplement: Figure S1 — The phylogenetic relationship of LCDs and DCDs in B. rapa with their related member in higher plants. NCBI accession numbers are NP_974843.1 for Arabidopsis thaliana LCD (AtLCD), AFS17242.1 for Brassica napus LCD (BnLCD), NP_175275.3 for Arabidopsis thaliana DCD1 (AtDCD1), NP_974363.1 for Arabidopsis thaliana DCD2 (AtDCD2), NP_001234368.1 for Solanum lycopersicum DCD (SlDCD), XP_007037066.1 for Theobroma cacao DCD1 (TcDCD1), XP_007037067.1 for Theobroma cacao DCD2 (TcDCD2), XP_007037068.1 for Theobroma cacao DCD3 (TcDCD3), and XP_003631148 for Medicago truncatula DCD (MtDCD). (TIF) [file pone.0110904.s001.tif]

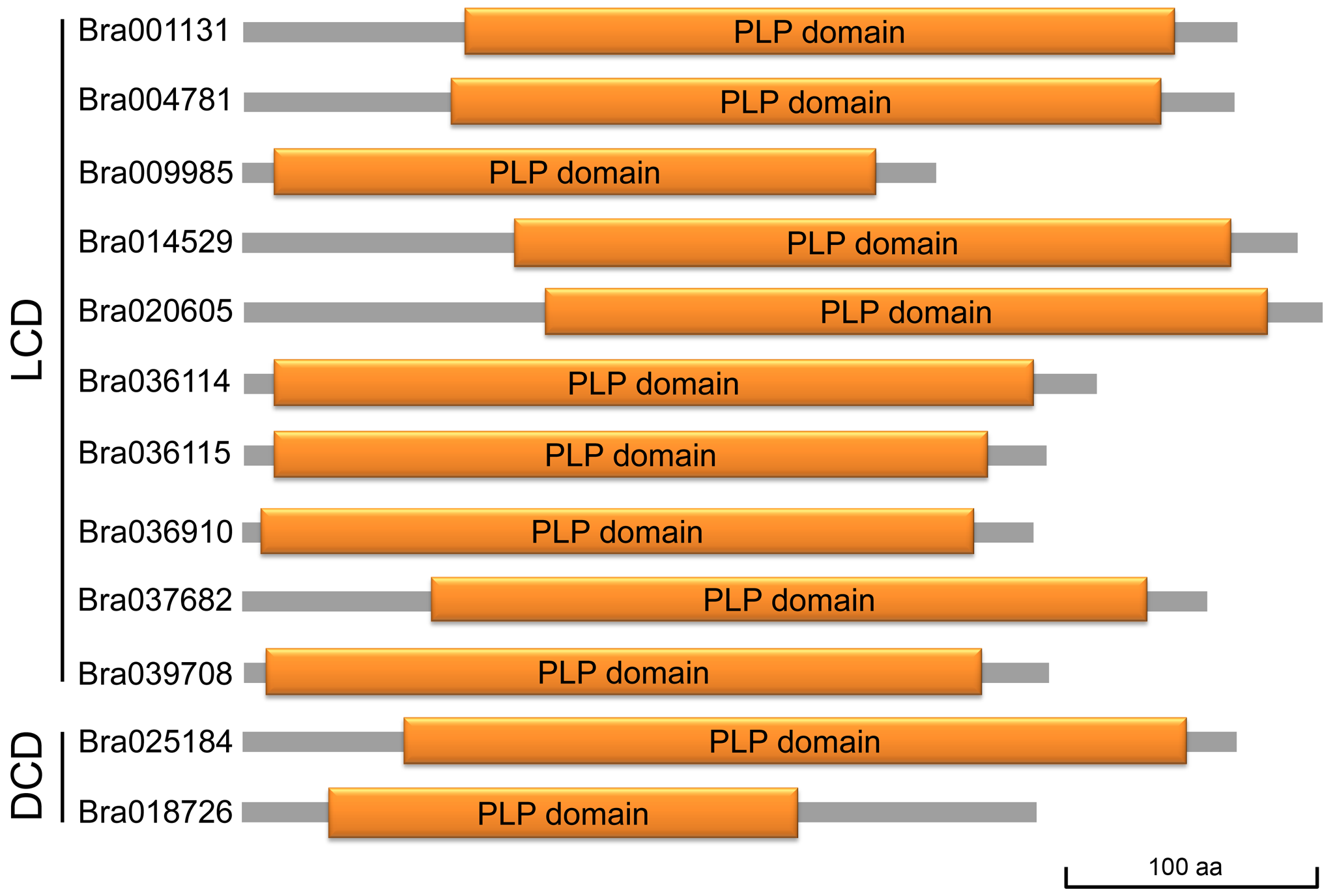

Supplement: Figure S2 — The location of PLP-dependent domain in LCDs and DCDs from B. rapa . The protein structure of two LCDs and two DCDs were analyzed by online tool SMART. The typical PLP-dependent domains were indicated as orange box. Bar indicated 100 amino acids (aa). (TIF) [file pone.0110904.s002.tif]

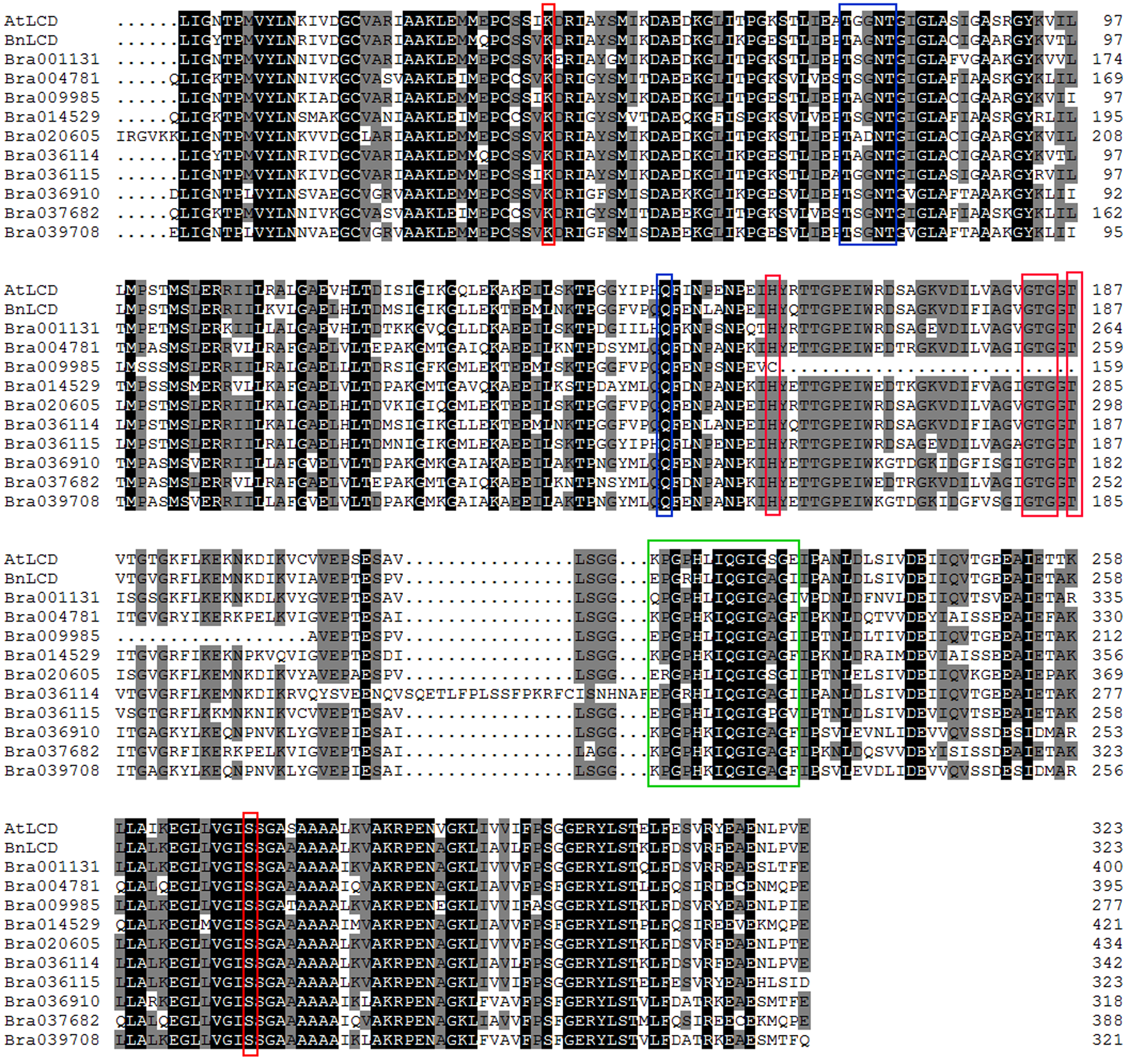

Supplement: Figure S3 — Alignment of the predicted amino acid sequences of LCDs in A. thaliana , B. napus , and B. rapa . Dark shading with white letters and gray shading with black letters reveal 100% and 75% sequence similarity, respectively. Database accession numbers are the same as described in Figure S1. The PLP-binding sites are shown by red box. The substrate binding site is indicated by blue box. The SAT protein-interaction site is indicated by red box. (TIF) [file pone.0110904.s003.tif]

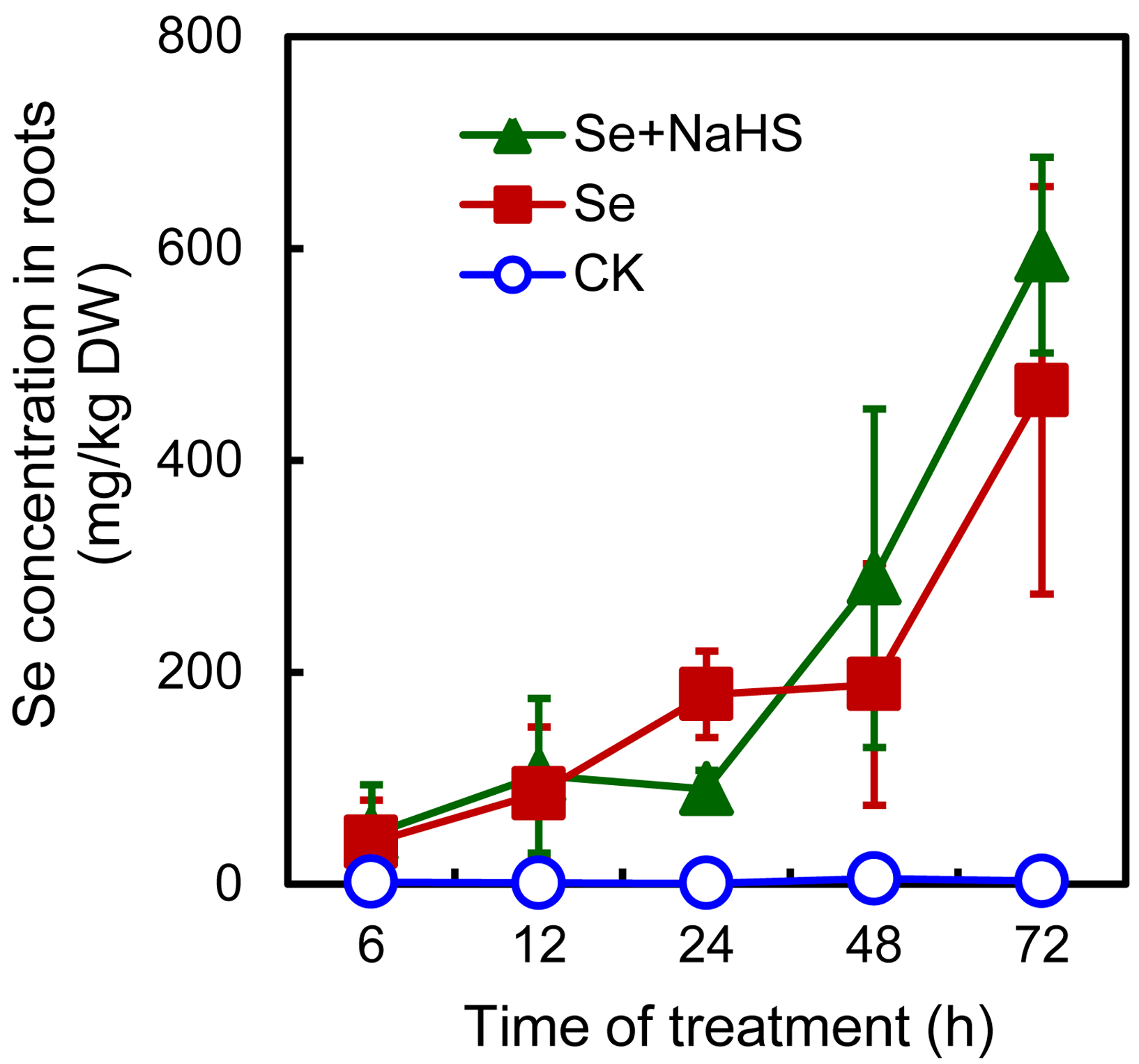

Supplement: Figure S4 — The concentration of Se in the roots of B. rapa exposed to Se(IV) or Se(IV)+NaHS. The roots were exposed to Se(IV) (0.06 mM) or Se(IV) (0.06 mM)+NaHS (0.5 mM) for 6, 12, 24, 48, 72 h, respectively. The roots were harvested at each point of treatment time for Se analysis. Each value was presented as the mean of three replicates with SD. (TIF) [file pone.0110904.s004.tif]
