# Supplementary material for: Selenium Inhibits Root Elongation by Repressing the Generation of Endogenous Hydrogen Sulfide in Brassica rapa
Source: PLoS One. 2014 Oct 21;9(10):e110904. doi: 10.1371/journal.pone.0110904 (PMC4204939; doi:10.1371/journal.pone.0110904)
Supplement: Table S1 — Sequences of oligonucleotide primers for qRT-PCR. F: forward; R: reverse. (DOCX) [file pone.0110904.s005.docx]

| **BRAD Number** | **Primer sequence (5’-3’)** |
| --- | --- |
| Bra018726 | **F:** CAAGGGAGATTGAGGAGCAG  **R:** TTCGGATCCTTGGTCATCTC |
| Bra025184 | **F:** CAGCAGCTTGGATACGATCA  **R:** ACCAAACATCCCAAGAGTGC |
| Bra001131 | **F:** ACCGGACAATTTGGATTTCA  **R:** GCCGTTTTGCCACCTTAATA |
| Bra037682 | **F:** CAGCCAATCCCAAGATTCAT  **R:** TTGTGAGGTCCTGGTTTTCC |
| Bra004781 | **F:** CCACGGAAAGTGCCATACTT  **R:** TTCAGGTCTCTTTGCCACCT |
| Bra014529 | **F:** GCGATTTCAAGTGAGGAAGC  **R:** TCCCGGATAGACTGGAACAG |
| Bra039708 | **F:** GGCAAGGCTTCTTGCTCTTA  **R:** CTTTCCTTGTGGCATCGAAT |
| Bra036910 | **F:** CCTGCTAACCCAAAGATCCA  **R:** TATAAAACCAGCGCCAATCC |
| Bra009985 | **F:** GCGGTGGAACCTACAGAGAG  **R:** GAGCAAGAAGCTTGGCTGTT |
| Bra020605 | **F:** GGATTGTTGGTGGGCATATC  **R:** GTCGGCAGATTCTCTGCTTC |
| Bra006115 | **F:** GCACTGGTGGAACGGTTAGT  **R:** GGTTGGAATGACACCAGGAC |
| Bra006114 | **F:** TGGTCCGGAGATATGGAGAG  **R:** CCTGGTTCTCCTCCACTGAG |
| *Actin* | **F:** CTATCCTCCGTCTCGATCTCGC  **R:** CTTAGCCGTCTCCAGCTCTTGC |

**Table S1.** Sequences of oligonucleotide primers for qRT-PCR. F: forward; R: reverse.
