# Supplementary material for: Selenium Inhibits Root Elongation by Repressing the Generation of Endogenous Hydrogen Sulfide in Brassica rapa
Source: PLoS One. 2014 Oct 21;9(10):e110904. doi: 10.1371/journal.pone.0110904 (PMC4204939; doi:10.1371/journal.pone.0110904)
Supplement: Table S2 — Distribution of cis -elements response to NO (NRE) and auxin (ARE) in the promote region of LCDs and DCDs in B. rapa . (DOCX) [file pone.0110904.s006.docx]

**Table S2.** Distribution of *cis*-elements response to NO (NRE) and auxin (ARE) in the promote region of *LCDs* and *DCDs* in *B. rapa*. The plant motifs were predicted based on the publicly available *cis*-acting regulatory elements database PLACE (<http://bioinformatics.psb.ugent.be/webtools/plantcare/html/>) as a reference. Forward sequence is indicated as (+) while the complementary sequence is indicated as (-). “N” means non-detective.

|  | | ***DCD*** | |  | ***LCD*** | | | | | | | | | |
| --- | --- | --- | --- | --- | --- | --- | --- | --- | --- | --- | --- | --- | --- | --- |
|  | | **Bra018726** | **Bra025184** |  | **Bra001131** | **Bra004781** | **Bra009985** | **Bra014529** | **Bra020605** | **Bra036114** | **Bra036115** | **Bra036910** | **Bra037682** | **Bra039708** |
| **ARE** | ARFAT | N | 103(+) |  | 151(-)  1140(-) | 379 (+)  579 (-)  728 (-) | 881 (-) | 1689 (-) | 1314 (-) | N | 965 (+)  998 (+)  1594 (+)  1883 (+) | 9 (-) | 136 (+)  393 (-) | 1791 (-) |
|  | ASF1MOTIFCAMCV | N | 605(-) |  | 438(+)  1945(+)  775(-) | N | 457 (+)  375 (-) | N | N | 1230 (+)  300 (-) | 673 (+) | 331 (+) | N | 899 (-)  1976 (-) |
|  | AUXREPSIAA4 | N | N |  | N | N | N | 339 (-) | N | N | N | N | N | N |
| **NRE** | ACGT box | N | N |  | 120(+) | N | N | N | N | N | N | N | N | N |
|  | IBOX | 1132(-) | N |  | N | N | N | N | 478 (-) | 466 (-) | N | 70 (+) | N | N |
|  | MYCL | N | N |  | N | N | N | N | N | N | N | N | N | N |
|  | WBOX | 1121(-) | 26(-)  589(-) |  | 1119(+)  142(-)  776(-)  1152(-) | 267 (+) | 456 (+)  376 (-)  691 (-)  716 (-)  898 (-) | 1076 (+)  137 (-)  1368 (-)  1941 (-)  1994 (- | 63 (+) | 284 (+)  786 (+)  960 (+)  1100 (+)  1121 (+)  1410 (+)  1580 (- | 672 (+)  812 (+)  1927 (+)  636 (-)  1400 (-) | 243 (+)  383 (+) | 37 (+) | 874 (+)  985 (+)  1443 (+)  656 (-)  677 (-)  1181 (-)  1496 (-) |
